# Whole exome sequencing reveals a de novo missense variant in *EEF1A2* in a Rett syndrome‐like patient

**DOI:** 10.1002/ccr3.2511

**Published:** 2019-11-12

**Authors:** Simranpreet Kaur, Nicole J. Van Bergen, Wendy Anne Gold, Stefanie Eggers, Sebastian Lunke, Susan M. White, Carolyn Ellaway, John Christodoulou

**Affiliations:** ^1^ Brain and Mitochondrial Research Group Murdoch Children's Research Institute Parkville Vic. Australia; ^2^ Department of Paediatrics University of Melbourne Parkville Vic. Australia; ^3^ Molecular Neurobiology Lab, Kids Research Westmead Children's Hospital Westmead NSW Australia; ^4^ Disciplines of Genetic Medicine and Child and Adolescent Health Sydney Medical School University of Sydney NSW Australia; ^5^ Translational Genomics Unit Murdoch Children's Research Institute Parkville Vic. Australia; ^6^ Victorian Clinical Genetics Services Murdoch Children's Research Institute Parkville Vic. Australia; ^7^ Genetic Metabolic Disorders Service Sydney Children's Hospital Network Sydney NSW Australia

**Keywords:** EEF1A2, elongation factor‐1, intellectual disability, mutation, Rett syndrome

## Abstract

Using whole exome sequencing, we found a pathogenic variant in the EEF1A2 gene in a patient with a Rett syndrome‐like (RTT‐like) phenotype, further confirming the association between EEF1A2 and Rett syndrome RTT and RTT‐like phenotypes.

## Graphical abstract

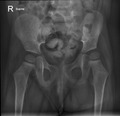

## 1 INTRODUCTION

Rett syndrome (RTT; OMIM 312750) is a severe, rare X‐linked neurodevelopmental disorder that primarily affects females and is notable for its progressive nature.^1^, ^2^ There are two major clinical categories of RTT syndrome: classical (or typical) RTT and atypical (or variant) RTT. Patients may also be classified as RTT‐like who do not meet the diagnostic criteria for a clinical diagnosis of classical or atypical RTT.^3^ In classical RTT, after a period of normal growth and development, patients exhibit distinct stages of disease. During stage I (6 ‐ 18 months of age), RTT females do not meet expected milestones due to developmental stagnation. Between 1 and 4 years (stage II) a rapid developmental regression phase features loss of acquired skills (eg, hand use and speech) with impaired social contact and patients develop stereotypic hand movements (wringing and washing). Microcephaly may become evident. Between 4 and 10 years (stage III) the clinical phenotype may stabilize, but affected individuals may develop breathing irregularities, seizures, and intense eye gaze. Beyond 10 years (stage IV), patients lose ambulation and experience muscle weakness, rigidity, spasticity, dystonia, and scoliosis; however, the hand stereotypic movements become less intense while eye contact and communication remain intact. Atypical RTT patients share many key of the supportive clinical features seen in classical RTT but do not have all of the main criteria.^3^ Almost all classical RTT patients (~98%) have pathogenic variants in the X‐linked Methyl CpG Protein 2 gene (*MECP2*),^4^ while only 60% of atypical RTT patients have pathogenic variants in *MECP2*.^5^

Although *MECP2* is the major causative gene for classical and atypical RTT, mutations in other genes including cyclin‐dependent kinase‐like 5 (*CDKL5*), Forkhead box protein G1 (*FOXG1*), myocyte‐specific enhancer factor 2C (*MEF2C*), and transcription factor 4 (*TCF4*) have also been found in patients with clinical profiles overlapping with RTT.^6^

Here, we report a RTT‐like patient who was negative for mutations in the more common genes associated with RTT. Previous genetic analyses failed to identify the causative gene. We identified a de novo heterozygous variant previously associated with intellectual disability but not RTT in the eukaryotic translation elongation factor 1 alpha 2 (*EEF1A2*: chr20 [q13.33]: 62,119,365‐62,130,668:11,304bp) gene.

## 2 CLINICAL REPORT

**Figure 1:**
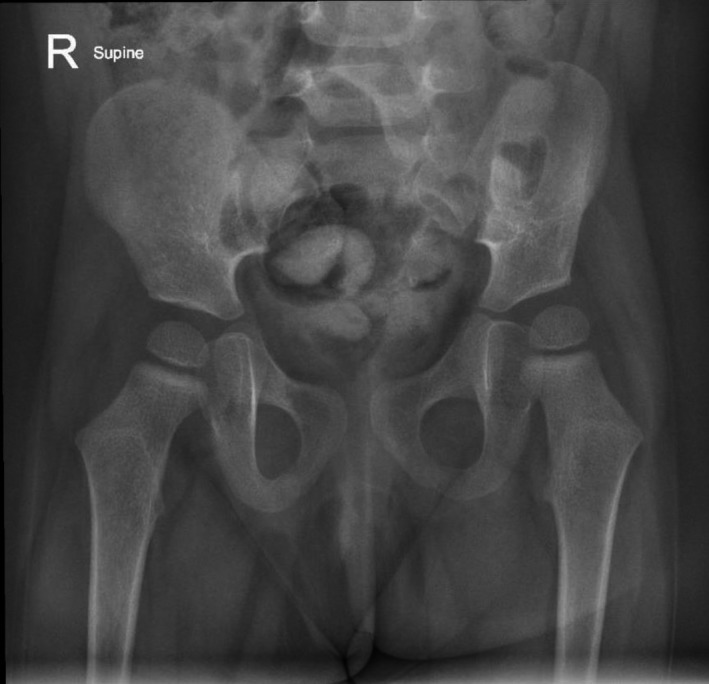
Pelvic X‐ray at 2 y of age showing resolution of the hip dysplasia

This study was approved by the Sydney Children's Hospitals Network Human Research Ethics Committee with written consent obtained from the parents. The proband was born at 39 weeks gestation following a pregnancy complicated by gestational diabetes managed with diet and normal delivery to nonconsanguineous parents. She flipped into a breech position at around 37 weeks and was delivered by cesarean section. Her Apgar scores were 9 at one and 9 at 5 minutes. Her birthweight was 2.75 kg (10th percentile), length was 50.5 cm (50th percentile), and head circumference was 31.5 cm (<2nd percentile). Blood sugar levels were monitored in the newborn period and were normal. She smiled at 6 weeks and was described as a sleepy baby. As she was breech, she had a routine hip scan at 6 weeks of age, which showed mild hip displacement, with a follow‐up scan at 4 months of age revealing hip dysplasia, which was managed in a Pavlik harness for 4 weeks. The hip dysplasia subsequently resolved, as evidenced by a normal pelvic X‐ray at 2 years of age (Figure 1). At 4 months of age, she was noted to have increased tone, scissoring of her legs and stared at her hands. At 6 months, she was thought to have a seizure. An EEG showed mild slowing for age but no epileptiform activity, while a head ultrasound was normal. At 14 months, she was seen by a Paediatric Neurologist who noted that she had small cold, hands and feet, was bringing her hands to her mouth, was babbling but not saying any words. The electroencephalogram and brain magnetic resonance imaging (MRI) were normal at this time. By 2 years and 3 months, her weight was 8.64 kg (2nd percentile), length was 72 cm (2nd percentile), and head circumference was 43 cm (<1st percentile). No facial dysmorphism was noticed by two experienced clinical geneticists. She was unable to roll or sit unsupported, articulate distinct words and had limited hand function. She had started to exhibit midline hand clasping movements and some hand‐to‐mouth movements. Her hands and feet were cold and sometimes mottled. She had feeding difficulties, constipation, and bruxism and was found to have central hypothyroidism. Seizures developed three months later. She had abnormal muscle tone and now exhibited an abnormal breathing pattern. She also had limited communicative abilities, inappropriate outbursts of laughter and a diminished response to pain. She could not sit unsupported and had poor head control. When last reviewed at 5 years of age, her weight was 14.3 kg (1st percentile), height approximately 100 cm (1st percentile), and head circumference was 46 cm (1st percentile). She continued to have episodes of breath‐holding and deep sighing respirations but no hyperventilation. She continued to grind her teeth, although not as frequently as previously. Her eye contact had improved. She had developed some additional stereotypic hand movements in the form of hand‐to‐mouth movements, sucking her knuckles, midline hand clasping, and tapping or rubbing her stomach. She had central hypotonia with increased tone of her limbs. Cardiovascular and respiratory examinations were normal. She also had a postural upper thoracic kyphosis and was able to stand with assistance.

## 3 GENETIC ANALYSIS

As part of the genetic diagnosis for the patient with RTT‐like features, sequencing of the *MECP2* gene at the Molecular Genetics Department, Children's Hospital at Westmead, did not identify any sequence variation and no deletion or duplication was detected by multiplex ligation‐dependent probe amplification studies. Subsequent genomic analysis of an epileptic encephalopathy gene set using the TruSight One sequencing panel at The Children's Hospital at Westmead, NSW, Australia, was also negative, although the coverage for some genes including *FOXG1* and *CDKL5* was not 100%. Singleton WES was therefore undertaken in order to try to identify the causative gene. WES SureSelectQXT Clinical Research Exome libraries were prepared and loaded onto a NextSeq 500 sequencer (Illumina; NextSeq control Software v2.1.031) and 2 × 150 bp paired‐end sequencing was performed at the Translational Genomics Unit, Victorian Clinical Genetic Services (VCGS). Samples passed sequencing QC with >89.7% bases with at least Q30, and mean coverage of at least 100‐fold. Raw sequencing data were converted to FASTQ format using Illumina's bcl2fastq2 converter (v2.17.1.14). Data were processed using Cpipe (http://cpipeline.org/),^7^ in order to generate annotated variant calls within the target region (coding exons ± 2bp), via alignment to the reference genome (GRCh37). Variants were annotated against all gene transcripts, with reporting of variants against the HGNC recommended transcript (according to HGVS nomenclature). Classification of variants was based on ACMG guidelines.^8^ A total of 25 679 variants were found via Cpipe analysis. All variants were uploaded onto Leiden Open (source) Variation Database (LOVD) prior to further analysis.^9^ First, the RTT associated genes were screened to examine any previously missed variants.^10^, ^11^ We then used a precurated phenotype‐specific gene list (https://www.vcgs.org.au/sites/default/files/media/TGW024_genelist_V3_0.pdf) provided by the VCGS for targeted analysis of known intellectual disability (ID) associated genes. This list consists of a total of 1,064 genes implicated in syndromic and nonsyndromic ID.

An unknown singleton inheritance filter was used to filter out variants with low quality (<100), genomic mean allelic frequency (>5%), and intronic splice region variants, untranslated region (UTR) variants, synonymous as well as low impact variants. The remaining 550 variants were further prioritized based upon the impact of the variant in the order of early stop gain, frameshift and missense variants. Pathogenicity of variants was evaluated using in silico tools including SIFT (http://sift.jcvi.org/), MutationTaster (http://www.mutationtaster.org/), PolyPhen‐2 (http://genetics.bwh.harvard.edu/pph2/), CADD, and Grantham scores.^12^, ^13^ The final shortlisted variants were then classified using The American College of Medical Genetics and Genomics (ACMG) guidelines 8 to interpret the consequence of the sequence variants. A literature search was conducted on each of the filtered variants to identify their implications in known disorders. In addition, 3‐dimensional molecular modeling was performed using the already published protein crystal structure through the automatic variant analysis server HOPE (http://www.cmbi.ru.nl/hope/).^14^

**Figure 2:**
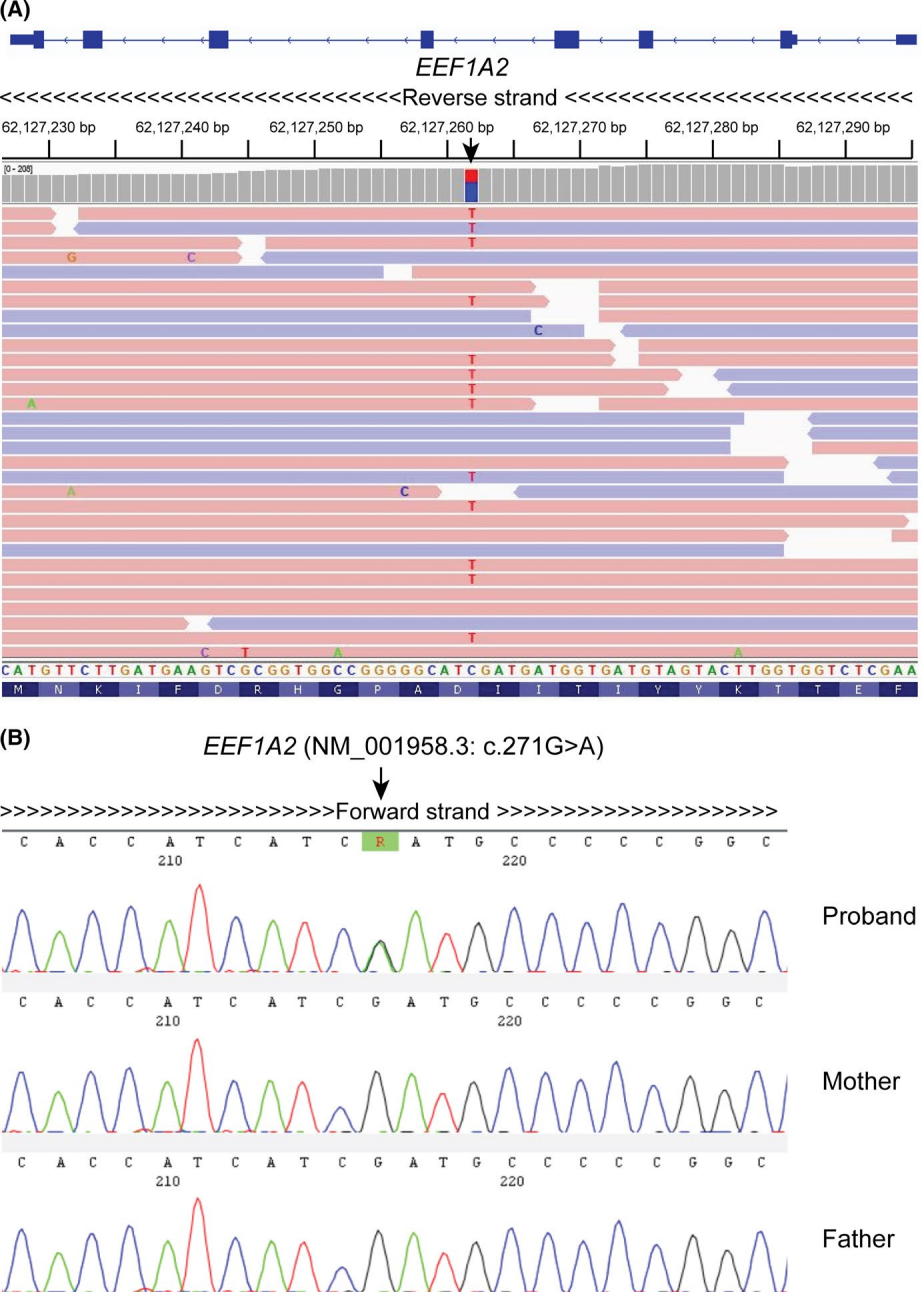
A, Schematic showing the Integrative Genomics Viewer reads (reverse strand) of whole exome sequencing encompassing the heterozygous variant (NM_001958.3 (*EEF1A2*): c.271G > A; p.(Asp91Asn)) reported in the proband. Note that *EEF1A2* is a reverse strand gene and hence the variant is shown as C > T change on the IGV reads. B, Sanger sequencing chromatogram (forward strand) showing a confirmed heterozygous variant for *EEF1A2* variant in the proband with both parents being homozygous wild type

**Figure 3:**
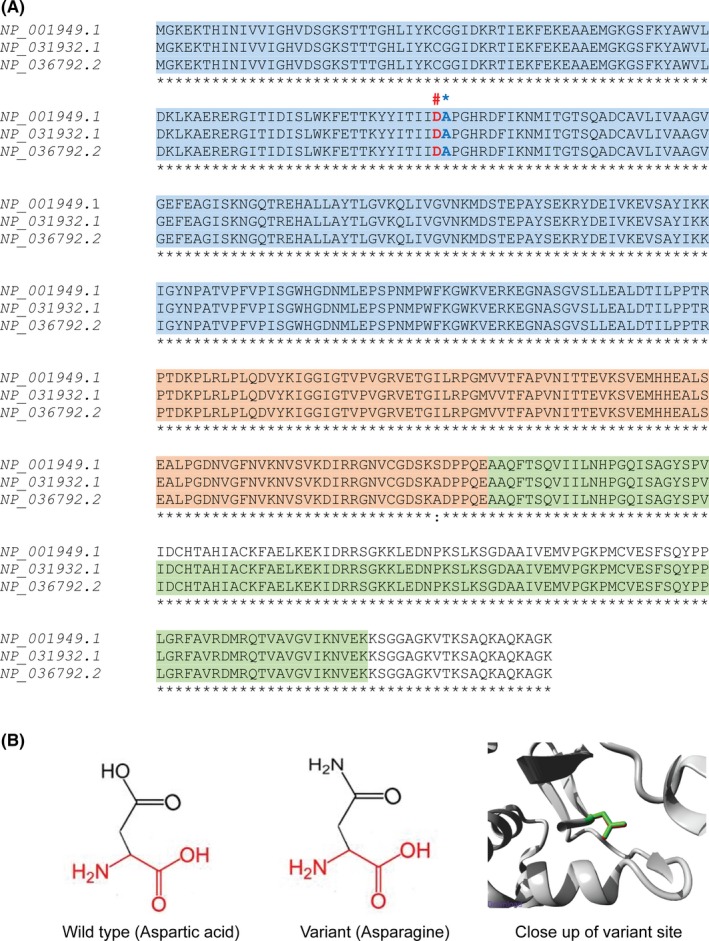
A, Evolutionary sequence conservation of EEF1A2. Schematics represents the multiple sequence alignment of EEF1A2 protein from human (NP_001949.1), mouse (NP_031932.1), and rat (NP_036792.2) performed using CLUSTAL O (1.2.4) (https://www.ebi.ac.uk/Tools/msa/clustalo/).The three structural domains are highlighted as domain I (residue 1‐240, highlighted blue), domain II (residue 241‐336, highlighted orange), and domain III (residue 337‐443, highlighted green). Domain I contains a helix that associates with the GTP and GDP. Domain I and domain II contain the eEF1B complex binding site for GTP/GDP exchange. Domain II and domain III harbor the aa‐tRNA binding site. Domain III contains an actin‐binding domain. The affected amino acid residue Asp91 (D) identified in our patient is marked (#) and the mutated residue Ala92 (A) described in Lopes, Barbosa et al (2016) is marked (*). B, Schematic structures of the wild type and variant amino acids with the close up of the variant site modeled with HOPE website. The protein is colored in gray, the side chain of the wild type and variant is green and red respectively. The backbone, which is the same for each amino acid, is colored red, the side chain, unique for each amino acid, is colored black

Using this filtering process, variants were prioritized based upon variant characteristics, in silico predictions of pathogenicity and expression in brain. We identified a heterozygous missense variant in *EEF1A2* (chr20: g.62127262C > T; NM_001958.3: c.271G > A; p.(Asp91Asn)) that has been previously reported 15 (Figure 2A). This variant was confirmed by Sanger sequencing DNA from child's parents to be de novo (Figure 2B). Based on multiple sequence alignment of the protein sequence using CLUSTAL O (1.2.4) (https://www.ebi.ac.uk/Tools/msa/clustalo/), the position of this aspartic acid is very highly conserved between different species including human (NP_001949.1), mouse (NP_031932.1), and rat (NP_036792.2) (Figure 3A). This variant is predicted to create an amino acid change from aspartic acid (Asp) to asparagine (Asn) at amino acid position 91 (Figure 3B). This variant was absent in population databases including Exome Aggregation Consortium (http://exac.broadinstitute.org ) and gnomAD (http://gnomad.broadinstitute.org/). In silico analysis predicted the variant to be damaging (SIFT, score 0.001), deleterious (PROVEN, score −3.85), and disease causing (MutationTaster) with a CADD Phred‐like score of 19.88 and Grantham score of 23. Based on the current in silico analysis, this variant has been classified as likely pathogenic based on ACMG criteria.

## 4 DISCUSSION

In this study, we have identified a de novo heterozygous variant in EEF1A2 (NM_001958.3: c.271G > A; p.(Asp91Asn)) in a patient with a RTT‐like phenotype. The identified NM_001958.3 (EEF1A2): c.271G > A substitution variant is predicted to change Asp to Asn at amino acid position 91, NP_001949.1 (EEF1A2): p.(Asp91Asn) affecting an evolutionarily conserved site in exon 3, and is situated within the guanosine triphosphate (GTP) binding domain. Heterozygous de novo mutations in EEF1A2 have previously been associated with neurodevelopmental disorders including epilepsy, autism, and severe intellectual disability.^15^, ^16^, ^17^, ^18^, ^19^, ^20^, ^21^, ^22^, ^23^, ^24^ Moreover, three siblings with dilated cardiomyopathy, global development delay, failure to thrive, epilepsy and ultimately death in early childhood have been reported to carry a homozygous mutation in EEF1A2 (NM_001958: c.1164C > T; p.Pro333Leu).^25^ The variant identified in our case has been previously reported as a de novo heterozygous variant in a 14‐year‐old female patient with, epilepsy, intellectual disability, hypotonia, cold peripheries, lack of speech, and inability to walk independently.^15^ Our patient shares some clinical features with the patient reported by Lam et al,^15^ including cold peripheries, seizures, hypotonia, and spine deformity. In addition, our patient also exhibits distinct features typically seen in RTT, including stereotypic hand movements, teeth grinding, limited speech, feeding difficulties, breath‐holding, inappropriate outbursts of laughter, a diminished response to pain, and no facial dysmorphism. This highlights that a variable neurodevelopmental phenotype may be associated with *EEF1A2* variants, with the same variant resulting in overlapping neurological presentations. Interestingly, a single case with a de novo heterozygous variant NM_001958: c.274G > A, p.(Ala92Thr) has also been reported in case of a 6‐year‐old RTT‐like female patient with stereotypic hand movements, loss of spoken language but no regression. She had seizures at 1 month of life and her development was significantly delayed, with delayed onset of speech (3 years) and ability to walk unaided (4 years). The patient also had hand stereotypies, bruxism, and crying spells when awake, sleep problems, hyperpnoea and apnea, and poor eye contact and was classified as RTT‐like.^18^

Three‐dimensional molecular modeling of the EEFA12 protein (Protein Data Bank code: 4C0S) performed using HOPE 14 predicted a change in the charge of the wild‐type (Asp; negative) to the mutant residue (Asn; neutral). Moreover, the wild‐type residue forms a hydrogen bond with Ala92 and Asn101, and a salt bridge with Arg67 26 which are predicted to be disrupted by the mutated residue. The difference in charge is likely to disturb ionic interactions created by the wild‐type residue and thus interfere with the conformational changes of the EEFA1A protein.

The eEF1A2 protein promotes the GTP‐dependent binding of aminoacyl‐tRNA to the A‐site of ribosomes during protein biosynthesis. During this process, eEF1A2 transits between an active GTP bound state and an inactive GDP bound state thus classified as Translational GTPase (trGTPases), a family of proteins in which GTPase activity of the protein is stimulated by the large ribosomal subunit. The other subunit, eEF1Bαβγ, then catalyses the nucleotide exchange of GDP for GTP in order to re‐activate the eEF1A complex for the next cycle of hydrolysis. Because of its functional role, binding, and hydrolysis of guanine (G) nucleotides, the G‐binding domain is a highly conserved between different species (Figure 3A). The amino acid Asp91 resides in this highly conserved domain and is stabilized by other amino acids when GDP is bound to the complex. This reaction is compromised during GDP displacement by the amino acid change we identified in this patient and consequently is predicted to affect the catalytic function of the protein. Because of the critical role of de novo protein synthesis machinery at the synaptic terminal, and the enrichment of eEF1 complex at the postsynaptic density region, the optimal functioning of the complex becomes critical for proper neuronal development. Any perturbation to this complex (in particular eEF1A2) is likely to result in neurodevelopmental dysfunction, as evidenced by affected individuals exhibiting varying clinical features including autism, epilepsy, and intellectual disability.^16^, ^17^, ^18^, ^25^, ^27^ Therefore, our in silico analysis supports this variant to be disease causing for this RTT‐like patient.

## 5 CONCLUSIONS

We have identified a de novo variant in *EEF1A2* in a patient with a Rett‐like phenotype that confirms the association between *EEF1A2* and a RTT‐like phenotype. This study also further emphasizes the clinical utility of singleton WES to identify causative genes in *MECP2* negative patients with RTT and RTT‐like phenotypes, and we suggest that analysis of *EEF1A2* should be included in the curation of genomic sequencing data from individuals with a RTT or RTT‐like clinical picture.

## CONFLICT OF INTEREST

None declared.

## AUTHOR CONTRIBUTIONS

SK: involved in design, experimental, analysis, and writing. NJVB and WAG: involved in design, analysis, and writing. SE and SL: involved in sequencing and writing. SMW and CJE: clinical input and writing. JC: design, analysis, writing, and overall oversight.

## References

[1] Rett A . On a unusual brain atrophy syndrome in hyperammonemia in childhood. Wien Med Wochenschr. 1966;116(37):723‐726. 5300597

[2] Hagberg B , Aicardi J , Dias K , Ramos O . A progressive syndrome of autism, dementia, ataxia, and loss of purposeful hand use in girls: Rett's syndrome: report of 35 cases. Ann Neurol. 1983;14(4):471‐479. 6638958 10.1002/ana.410140412

[3] Neul JL , Kaufmann WE , Glaze DG , et al. Rett syndrome: revised diagnostic criteria and nomenclature. Ann Neurol. 2010;68(6):944‐950. 21154482 10.1002/ana.22124 PMC3058521

[4] Percy AK , Lane J , Annese F , Warren H , Skinner SA , Neul JL . When Rett syndrome is due to genes other than MECP2. Transl Sci Rare Dis. 2018;3(1):49‐53. 29682453 10.3233/TRD-180021 PMC5900556

[5] Dolce A , Ben‐Zeev B , Naidu S , Kossoff EH . Rett syndrome and epilepsy: an update for child neurologists. Pediatr Neurol. 2013;48(5):337‐345. 23583050 10.1016/j.pediatrneurol.2012.11.001

[6] Armani R , Archer H , Clarke A , et al. Transcription factor 4 and myocyte enhancer factor 2C mutations are not common causes of Rett syndrome. Am J Med Genet A. 2012;158A(4):713‐719. 22383159 10.1002/ajmg.a.34206

[7] Sadedin SP , Dashnow H , James PA , et al. Cpipe: a shared variant detection pipeline designed for diagnostic settings. Genome Med. 2015;7(1):68. 26217397 10.1186/s13073-015-0191-x PMC4515933

[8] Richards S , Aziz N , Bale S , et al. Standards and guidelines for the interpretation of sequence variants: a joint consensus recommendation of the American College of Medical Genetics and Genomics and the Association for Molecular Pathology. Genet Med. 2015;17(5):405‐424. 25741868 10.1038/gim.2015.30 PMC4544753

[9] Fokkema IF , Taschner PE , Schaafsma GC , Celli J , Laros JF , den Dunnen JT . LOVD v.2.0: the next generation in gene variant databases. Hum Mutat. 2011;32(5):557‐563. 21520333 10.1002/humu.21438

[10] Christodoulou J , Grimm A , Maher T , Bennetts B . RettBASE: The IRSA MECP2 variation database‐a new mutation database in evolution. Hum Mutat. 2003;21(5):466‐472. 12673788 10.1002/humu.10194

[11] Krishnaraj R , Ho G , Christodoulou J . RettBASE: Rett syndrome database update. Hum Mutat. 2017;38(8):922‐931. 28544139 10.1002/humu.23263

[12] Adzhubei IA , Schmidt S , Peshkin L , et al. A method and server for predicting damaging missense mutations. Nat Methods. 2010;7(4):248‐249. 20354512 10.1038/nmeth0410-248 PMC2855889

[13] Kumar P , Henikoff S , Ng PC . Predicting the effects of coding non‐synonymous variants on protein function using the SIFT algorithm. Nat Protoc. 2009;4(7):1073‐1081. 19561590 10.1038/nprot.2009.86

[14] Venselaar H , Te Beek TA , Kuipers RK , Hekkelman ML , Vriend G Protein structure analysis of mutations causing inheritable diseases. An e‐Science approach with life scientist friendly interfaces. BMC Bioinformatics. 2010;11:548. 21059217 10.1186/1471-2105-11-548 PMC2992548

[15] Lam W , Millichap JJ , Soares DC , et al. Novel de novo EEF1A2 missense mutations causing epilepsy and intellectual disability. Mol Genet Genomic Med. 2016;4(4):465‐474. 27441201 10.1002/mgg3.219 PMC4947865

[16] Veeramah KR , Johnstone L , Karafet TM , et al. Exome sequencing reveals new causal mutations in children with epileptic encephalopathies. Epilepsia. 2013;54(7):1270‐1281. 23647072 10.1111/epi.12201 PMC3700577

[17] Nakajima J , Okamoto N , Tohyama J , et al. De novo EEF1A2 mutations in patients with characteristic facial features, intellectual disability, autistic behaviors and epilepsy. Clin Genet. 2015;87(4):356‐361. 24697219 10.1111/cge.12394

[18] Lopes F , Barbosa M , Ameur A , et al. Identification of novel genetic causes of Rett syndrome‐like phenotypes. J Med Genet. 2016;53(3):190‐199. 26740508 10.1136/jmedgenet-2015-103568

[19] Lelieveld SH , Reijnders MRF , Pfundt R , et al. Meta‐analysis of 2,104 trios provides support for 10 new genes for intellectual disability. Nat Neurosci. 2016;19(9):1194‐1196. 27479843 10.1038/nn.4352

[20] Iossifov I , O'Roak BJ , Sanders SJ , et al. The contribution of de novo coding mutations to autism spectrum disorder. Nature. 2014;515(7526):216‐221. 25363768 10.1038/nature13908 PMC4313871

[21] Inui T , Kobayashi S , Ashikari Y , et al. Two cases of early‐onset myoclonic seizures with continuous parietal delta activity caused by EEF1A2 mutations. Brain Dev. 2016;38(5):520‐524. 26682508 10.1016/j.braindev.2015.11.003

[22] Epi K , Bellows ST , Berkovic SF , et al. Ultra‐rare genetic variation in common epilepsies: a case‐control sequencing study. Lancet Neurol. 2017;16(2):135‐143. 28102150 10.1016/S1474-4422(16)30359-3

[23] de Ligt J , Willemsen MH , van Bon BWM , et al. Diagnostic exome sequencing in persons with severe intellectual disability. N Engl J Med. 2012;367(20):1921‐1929. 23033978 10.1056/NEJMoa1206524

[24] Ohba C , Haginoya K , Osaka H , et al. De novo KIF1A mutations cause intellectual deficit, cerebellar atrophy, lower limb spasticity and visual disturbance. J Hum Genet. 2015;60(12):739‐742. 26354034 10.1038/jhg.2015.108

[25] Cao S , Smith LL , Padilla‐Lopez SR , et al. Homozygous EEF1A2 mutation causes dilated cardiomyopathy, failure to thrive, global developmental delay, epilepsy and early death. Hum Mol Genet. 2017;26(18):3545‐3552. 28911200 10.1093/hmg/ddx239 PMC5886049

[26] Crepin T , Shalak VF , Yaremchuk AD , et al. Mammalian translation elongation factor eEF1A2: X‐ray structure and new features of GDP/GTP exchange mechanism in higher eukaryotes. Nucleic Acids Res. 2014;42(20):12939‐12948. 25326326 10.1093/nar/gku974 PMC4227793

[27] de Kovel CG , Brilstra EH , van Kempen MJA , et al. Targeted sequencing of 351 candidate genes for epileptic encephalopathy in a large cohort of patients. Mol Genet Genomic Med. 2016;4(5):568‐580. 27652284 10.1002/mgg3.235 PMC5023942

